# Detection of RHDV strains in the Iberian hare (*Lepus granatensis*): earliest evidence of rabbit lagovirus cross-species infection

**DOI:** 10.1186/s13567-014-0094-7

**Published:** 2014-09-24

**Authors:** Ana M Lopes, Sara Marques, Eliane Silva, Maria J Magalhães, Ana Pinheiro, Paulo C Alves, Jacques Le Pendu, Pedro J Esteves, Gertrude Thompson, Joana Abrantes

**Affiliations:** CIBIO-UP, Centro de Investigação em Biodiversidade e Recursos Genéticos-Universidade do Porto/InBIO, Laboratório Associado, Vairão, Portugal; Departamento de Biologia, Faculdade de Ciências da Universidade do Porto, Porto, Portugal; INSERM, UMR892; CNRS, UMR6299, Université de Nantes, Nantes, France; ICBAS, Departamento de Clínicas Veterinárias, Instituto de Ciências Biomédicas Abel Salazar, Universidade do Porto, Porto, Portugal; SaBIO – Instituto de Investigacion en Recursos Cinegeticos (CSIC-UCLM-JCCM), Ciudad Real, Spain; Wildlife Biology Program, University of Montana, 32 Campus Drive, Missoula, Montana 59812 USA; Instituto de Investigação e Formação Avançada em Ciências e Tecnologias da Saúde (CESPU), Gandra, Portugal

## Abstract

Rabbit hemorrhagic disease virus (RHDV) is a highly lethal *Lagovirus*, family *Caliciviridae*, that threatens European rabbits (*Oryctolagus cuniculus*). Although a related virus severely affects hares, cross-species infection was only recently described for new variant RHDV in Cape hares (*Lepus capensis mediterraneus*). We sequenced two strains from dead Iberian hares (*Lepus granatensis*) collected in the 1990s in Portugal. Clinical signs were compatible with a *Lagovirus* infection. Phylogenetic analysis of the complete capsid gene positioned them in the RHDV genogroup that circulated on the Iberian Peninsula at that time. This is the earliest evidence of RHDV affecting a species other than European rabbits.

## Introduction, methods and results

Wild and domestic European rabbits (*Oryctolagus cuniculus*) are severely affected by rabbit hemorrhagic disease (RHD), an acute hepatitis that causes death within 48–72 h after infection (reviewed in [1]). RHD was first reported in 1984 in China and two years later was described in Italy, spreading to several European countries afterwards (reviewed in [1]). On the Iberian Peninsula (IP), the first reports of RHD date from 1988 (reviewed in [1]). The etiological agent of the disease, rabbit hemorrhagic disease virus (RHDV), belongs to the *Caliciviridae* family, genus *Lagovirus*. The other member of this genus, European brown hare syndrome virus (EBHSV), causes the European brown hare syndrome (EBHS) in European and mountain hares (*Lepus europaeus* and *L. timidus*, respectively) [2]. On the IP, EBHSV was first reported in *L. europaeus* in 1995 (Gortázar C, personal communication). RHDV and EBHSV are non-enveloped, positive-sense, single-stranded RNA viruses, with a similar genomic organization and ~70% of nucleotide identity [3]. RHD and EBHS share clinical signs and pathological alterations, including congestion and necrosis of the liver and massive disseminated intravascular coagulation (reviewed in [1]).

**Figure 1:**
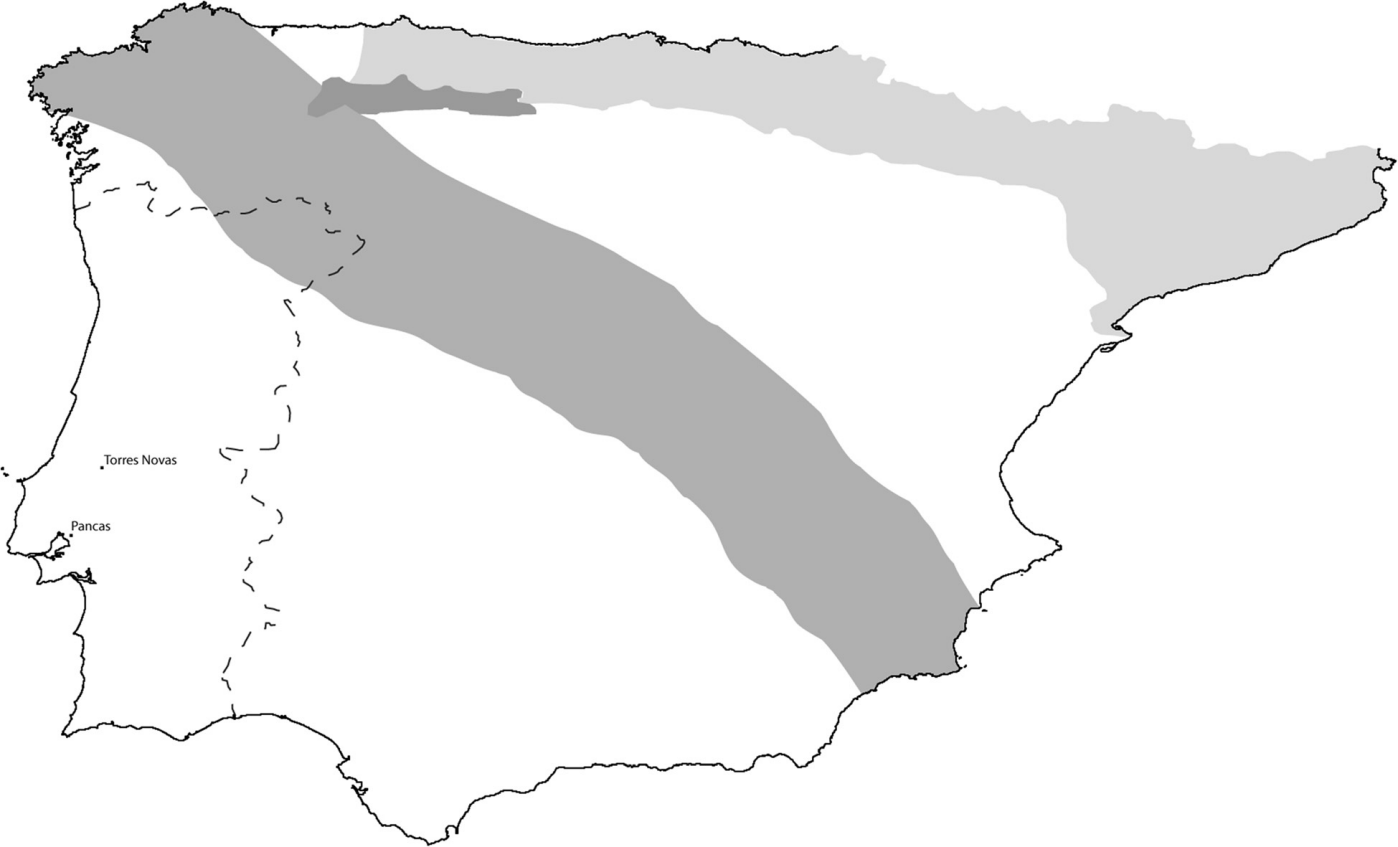
**Map with the distribution of leporid species on the Iberian Peninsula.** Light grey represents the distribution of *Lepus europaeus*. Dark grey corresponds to the distribution of *L. castroviejoi*. White represents the distribution of *L. granatensis*. The European rabbit (*Oryctolagus cuniculus*) is present all over the Iberian Peninsula; the contact zone between *O. c. cuniculus* and *O. c. algirus* (restricted to the southwest region) is in medium grey. The localities where P95 (Torres Novas) and P151 (Pancas) were collected are indicated.

Two subspecies of *O. cuniculus* are found on the IP, *O. cuniculus cuniculus* in the northeast and *O. cuniculus algirus* in the southwest [4] (Figure 1). Additionally, three hare species inhabit this region: *L. granatensis*, distributed along Portugal, mainland Spain and Majorca; *L. castroviejoi*, restricted to Cantabria; and *L. europaeus*, confined to the Pyrenees [4] (Figure 1). Although EBHS was reported in Spain, there are no reports of the disease in *L. granatensis* and *L. castroviejoi*, both endemic to the IP.

Despite their similarities, RHDV and EBHSV are restricted to their natural hosts, as has also been proven experimentally [5]; however, cross infection was recently described for a new RHDV variant (tentatively named RHDV2 or RHDVb) in *Lepus capensis mediterraneus* in Italy [6]. RHDV2 was originally described in France in 2010 [7], and is antigenically and genetically different from the classical RHDV strains [7,8]. In this study we report the first and earliest evidence of cross-species infection of classical RHDV in the Iberian hare, *L. granatensis*.

Liver samples of two hares found dead in the field were collected in Torres Novas and Pancas, south of Portugal, in 1996 and 1998, respectively (P95 and P151; Figure 1). At necropsy, lesions compatible with an infection by a *Lagovirus* were observed. P95 showed congestion of the liver and lungs as well as non-coagulated blood in the chest cavity, trachea and lungs; P151 presented congestion of the kidneys and both the chest and abdominal cavity were filled with non-coagulated blood and presented dark red coloration. In both animals, blood vessels were dilated and filled with non-coagulated blood. Additionally, the livers of five dead rabbits collected in Pancas in November and December-1997 were included in this study (P129, P141-P144). These individuals also exhibited lesions compatible with a *Lagovirus* infection at necropsy: dark red coloration and congestion of the kidneys and spleen; heart in diastole and atria filled with blood; non-coagulated blood in the trachea, chest and abdominal cavity (P143 presented normal abdominal cavity). With the exception of P129, dilated blood vessels filled with non-coagulated blood were observed, as well as a hypertrophic, congested liver. P142-P144 additionally presented congested lungs, filled with non-coagulated blood and P129, P141 and P142 presented nasal hemorrhages.

To avoid any risk of contamination, RNA extraction, cDNA synthesis and PCR amplification of the two hare samples were performed independently from the other samples. Total RNA was extracted with the RNeasy Mini Kit (Qiagen, Hilden, Germany) and reverse transcription was performed using oligo(dT) as primers and SuperScript™ III Reverse Transcriptase (Invitrogen, Carlsbad, CA, USA). Protocols were performed according to the manufacturer’s instructions. The samples were screened for the presence of RHDV and EBHSV by PCR with the primers U38 and EBHS9 as described elsewhere [9] as these primers amplify both RHDV and EBHSV. Sequencing of the fragments revealed that samples were positive for RHDV and negative for EBHSV. Additionally, and to ensure that only RHDV was present, RHDV and EBHSV-specific PCR were also performed (conditions available upon request). Amplification was successful for RHDV, but not for EBHSV. The gene encoding the capsid protein VP60 of RHDV (1740 base pairs) was fully amplified by PCR using 1 μL of the cDNA reaction, 2 pmol of each oligonucleotide, 5 μL of Phusion Flash High-Fidelity PCR Master Mix (Thermo Scientific, Waltham, Massachusetts, USA) and water to a final volume of 10 μL, using several pairs of primers (PCR cycle conditions are available upon request). After purification, PCR products were sequenced on an automatic sequencer ABI PRISM 310 Genetic Analyzer (PE Applied Biosystems, Foster City, CA, USA) with the amplification primers. Sequences were submitted to GenBank under the accession numbers KJ943791 and KJ943792 for the sequences from hares and KJ943793-KJ943797 for the sequences from rabbits. A Blast analysis of the sequences revealed 98% identity of P95 with strain AST89 (genogroup 1; accession number Z49271) and 97% identity of P151 with strain 00–08 (Iberian group 3 of genogroup 1 [10]; accession number AJ319594).

**Figure 2:**
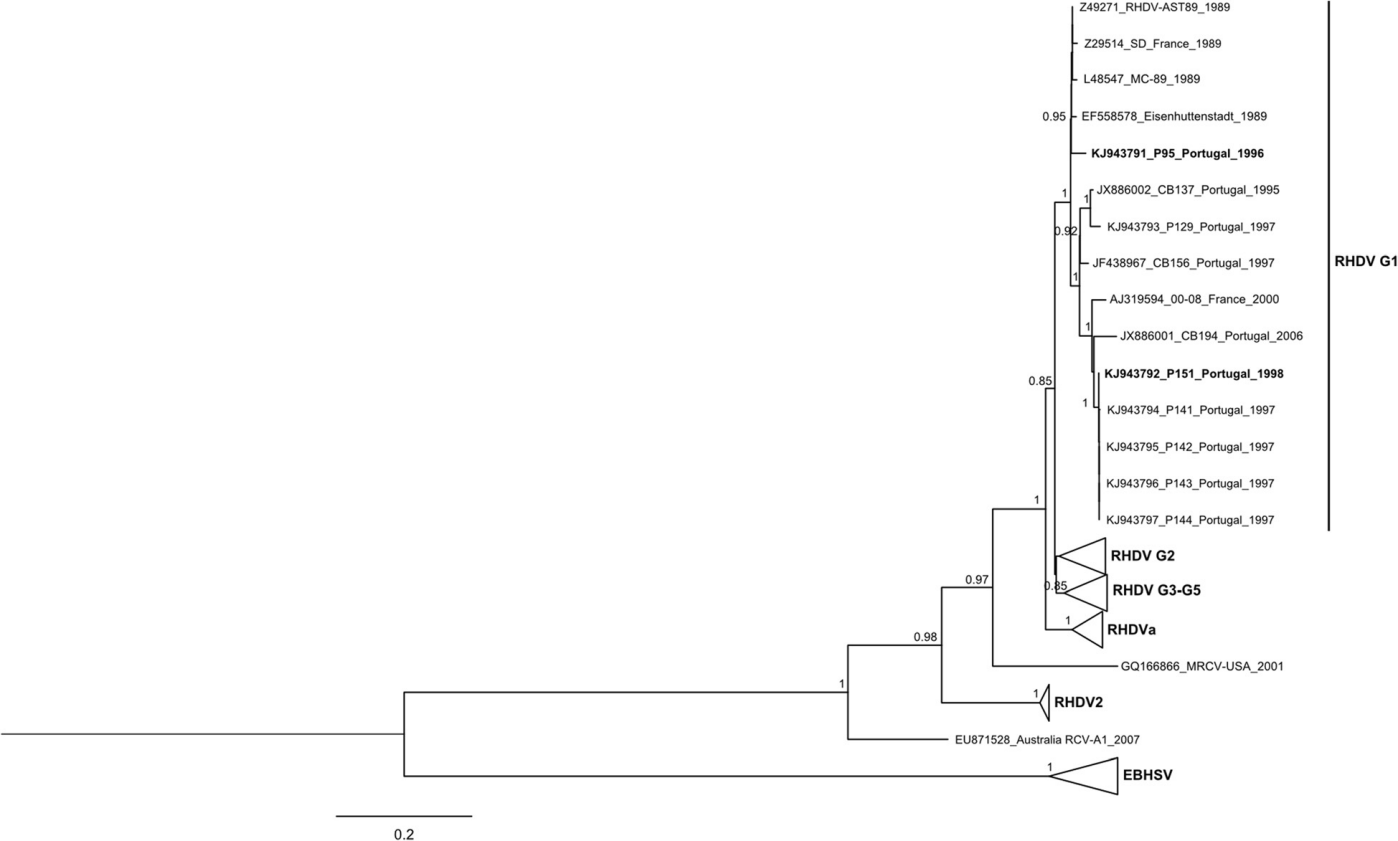
**Maximum Likelihood (ML) tree of the capsid gene VP60 for** ***Lagovirus*** **.** Only bootstrap values ≥ 0.85 are shown. In order to facilitate visualization, major groups are collapsed, with the exception of the group containing the specimens analyzed in the study. The sequences retrieved from GenBank database for this analysis have the following accession numbers: KF494918, KF494921, KF494922, KF494924, KF494930, KF494932, KF494936, KF494943, KF494947, KF494950, KF494951, GU373618, EF558580, M67473, EU650679, EU003579, U49726, AF295785, U54983, FR823355, FJ212323, AF402614, EU003580, EF558579, JN851735, AF231353, DQ189078 (sequences from G2); AM085133, EF558576, EF558572, Y15424, EF558574, AJ535094, AJ535092, Y15426, X87607, FR823354, EF558577, EF558585, Y15441, EF558575, KC595270, AY928268, DQ189077 (sequences from G3-G5); AY523410, KF270630, AF258618, EU003582, DQ205345, DQ280493, AB300693, HM623309, EF558581, EU003578, EU003581, EF558583, EF558582, EF558584, JF412629 (RHDVa sequences); HE800529, FR819781, HE800532, HE800531, HE819400, HE800530, KC345612, JQ929052, KC345611 (RHDV2); Z32526, X98002, U09199, KC832838, KC832839, AM408588, AM933648, AM933649, AM933650, AM887765 (EBHSV sequences). RHDV sequences from Iberian hares appear in bold.

The capsid sequences were aligned with publicly available sequences of *Lagovirus* (for simplicity, only part of the available sequences were used), namely RHDV genogroups 1–5 (G1-G5) [11], the antigenic variant RHDVa (G6), RHDV2, the weakly pathogenic MRCV, the non-pathogenic strain RCV-A1, and EBHSV (accession numbers of the sequences used are indicated in Figure 2). Furthermore, the alignment was screened for recombination as described elsewhere [12]. The phylogenetic relationships between the samples were estimated in MEGA6 [13], by using a Maximum Likelihood (ML) approach. The support of the nodes was assessed with a bootstrap resampling analysis (1000 replicates). The best-fit nucleotide substitution model defined by the same software (GTR + I + G) was used in the analysis.

The inferred ML relationships were in agreement with those published, with EBHSV, RHDV2 and RHDVa forming well supported groups (bootstrap values of 1; Figure 2). Genogroups 1–5 cluster together, with G1, G2 and G3-G5 also forming well supported subgroups. The tree further shows the inclusion of P95 and P151 in the G1 subgroup: P151 clusters with the rabbit samples collected in the same area (P129 and P141-P144), with the Portuguese strains CB137, CB156 and CB194, dating from 1995, 1997 and 2006, respectively, and with the French strain 00–08 dating from 2000; P95 appears to be more closely related to the older European strains belonging to G1, from Spain, France and Germany. The higher average nucleotide and amino acid identity of P95 and P151 is found with G1 (95.9%-96.6% of nucleotide identity and 97.8%-98.4% of amino acid identity). Furthermore, P151 presents 99.8%-99.9% of nucleotide identity and 100% of amino acid identity with P141-P144.

In order to confirm the species identity of P95 and P151, one mitochondrial (cytochrome *b*) and three nuclear markers (*CCR5*, *CXCR4* and CH2 domain of *IGHG*) were amplified for both samples. Total genomic DNA was extracted from the liver samples using the EasySpin Genomic DNA Minipreps Tissue Kit (Citomed, Lisbon, Portugal) according to the manufacturer’s instructions. The nuclear markers have been shown to present specific SNP for *Oryctolagus* and *Lepus* and their amplification followed the conditions described in [14-16]. The amplification of the mitochondrial marker was performed using the primers described in [17]. PCR was performed in a final volume of 10 μL containing 1 μL of DNA, 3 pmol of each primer and 5 μL of HotStartTaq DNA Polymerase Multiplex PCR Master Mix (Qiagen) (PCR cycle conditions are available upon request). After purification, PCR products were sequenced with the amplification primers as described above. Sequences of P95 and P151 for all markers were deposited in GenBank under the accession numbers KJ943798-KJ943805. The obtained sequences were then visually inspected and aligned, and compared with publicly available sequences of lagomorphs. Genetic distances between P95 and P151 and the other lagomorph sequences were calculated for each marker in MEGA6 [13], using the p-distance method and 500 replicates; all positions with less than 95% site coverage were eliminated. Additionally, an ML approach was also used to confirm the phylogenetic position of P95 and P151 in the phylogeny of lagomorphs for each marker, using the best model estimated for each marker (Kimura2 + G for *CCR5* and *CXCR4*; T92 for *IGHG*-CH2 and GTR + I + G for cytochrome *b*) and 500 replicates. The analysis of the genetic distances between P95 and P151 and other lagomorphs indicates that both specimens are closer to *L. granatensis* for all markers (genetic distance of 0.0% for nuclear genes and 0.6% for the mitochondrial gene; Table 1). Despite the lack of information for some markers, the overall highest genetic distance was observed for *Ochotona* spp: 8.4%-18% for nuclear markers and 19.9%-20.3% for cytochrome *b*. The phylogenetic analysis performed for each marker also supports the clustering of P95 and P151 with *L. granatensis* (data not shown).

**Table 1:** **Genetic distances of P95 and P151 relative to lagomorphs for nuclear markers** ***CCR5*** **,** ***CXCR4*** **and** ***IGHG*** **-CH2 and mitochondrial marker cytochrome** ***b***

	***CCR5***		***CXCR4***		***IGHG*** **-CH2**		**cytochrome** ***b***	
	**P95**	**P151**	**P95**	**P151**	**P95**	**P151**	**P95**	**P151**
*Lepus granatensis*	0.0	0.0	0.0	0.0	0.0	0.0	0.6*	0.6*
*Lepus europaeus*	0.2	0.2	0.5	0.5	0.9	0.9	8.1*	8.1*
*Lepus timidus*	0.2	0.2	-	-	0.0	0.0	9.7*	9.7*
*Lepus americanus*	-	-	0.4	0.4	0.9	0.9	9.8*	9.8*
*Lepus callotis*	2.7	2.7	-	-	1.1	1.1	9.1*	9.1*
*Lepus californicus*	-	-	0.3	0.3	1.4	1.4	9.7*	9.7*
*Lepus townsendii*	0.4	0.4	0.3	0.3	-	-	9.4*	9.4*
*Lepus capensis*	-	-	-	-	1.1	1.1	9.7*	9.7*
*Lepus saxatilis*	0.0	0.0	0.3	0.3	1.1	1.1	9.8*	9.8*
*Lepus castroviejoi*	0.4	0.4	0.4	0.4	0.9	0.9	10.9*	10.8*
*Sylvilagus brasiliensis*	2.3	2.3	1.1	1.1	-	-	-	-
*Sylvilagus floridanus*	2.9	2.9	0.8	0.8	-	-	15.9*	16.1*
*Bunolagus monticularis*	5.1	5.1	1.0	1.0	-	-	14.3*	14.5*
*Oryctolagus cuniculus*	4.9*	4.9*	1.1*	1.1*	4.2*	4.2*	14.7*	14.9*
*Ochotona princeps*	18.0	18.0	8.9	8.9	-	-	19.9*	20.1*
*Ochotona dauurica*	-	-	8.4	8.4	-	-	20.2*	20.3*

Only species with information for at least two markers are presented in the table. Values are given in percentages; “-” means lack of information and “*” means average value (obtained by the mean of the genetic distances of three randomly selected sequences for that species).

GenBank accession numbers: DQ146764, DQ146763, GU047870, GU047868, GU047869, GU047871, GU047867, GU047872, DQ017768, GU047865, AM051305, AM051298, DQ017767, GU047873 (*CCR5*); EU258270, EU258265, EU258269, EU258271, EU258266, EU258268, EU258267, EU258273, EU258272, EU258264, EU258287, EU258292, EU258304, EU258275, EU258274 (*CXCR4*); AJ295217, AJ295221, AJ295216, AJ431716, AJ295220, AJ295219, AJ295218, AJ295222, AJ431717, L29172, AY386696, AJ430862 (*IGHG*-CH2); AY942565, HQ596476, AF157465, AY745113, HQ596473, AJ421471, HM233015, AY599076, AB687529, KF781432, KF781431, KF781430, HQ596467, AF010159, HQ596468, KF781354, HQ596464, KF781357, HQ596485, JN037375, JN037374, HM233008, AY292732, EU729261, HQ596480, AF009731, AY292730, AY942569, JN037350, AY176235, AY292724, U58939, AF034257, AY292718, U58931, AY292717, HG810783, HG810780, EU591094, EU591077, EU590965, AF273000, JF911809, EF567059 (cytochrome *b*).

## Discussion

Evolution of RHDV has been widely characterized, with strains grouping according to their year of isolation [10-12,18,19]. In France, strains are divided into six genogroups, G1-G6, and most genogroups have been successively replaced [11]. Interestingly, G1 was able to persist only in the Iberian Peninsula (IP) [10] and, until 2011, it was the only genogroup found, most likely due to the Pyrenees acting as a major natural barrier that limits virus dispersal [10]. The antigenic variant RHDVa was also detected, but only in farms and never in wild populations [20]. From 2011, the new variant RHDV2 or RHDVb was detected and G1 seems to have been replaced [8,21,22]. Interestingly, RHDV2 was recently reported in Cape hares, *L. c. mediterraneus* [6], constituting the first evidence of cross infection in *Lagovirus*. Here we amplified and sequenced the complete capsid protein gene VP60 of a *Lagovirus*, confirmed to be RHDV, from Iberian hares found dead in the field in the 1990s in Portugal.

The phylogenetic analysis of the complete capsid gene VP60 revealed that the sequences obtained from the two Iberian hare specimens group with RHDV genogroup 1 (Figure 2). Since G1 was the only genogroup known to circulate in IP at the time of sample collection (1996 and 1998), this supports our finding of a cross-species infection of RHDV. Additionally, it appears that two different strains were able to infect hares: the sequences of P95 and P151 present 96 nucleotide differences that translate into 17 amino acid differences in the VP60 (nucleotide genetic distance of 6% and amino acid genetic distance of 2.8%; data not shown). Moreover, the strains are located in different nodes of the phylogenetic tree: P151 clusters with a recent French strain and Portuguese strains, including RHDV strains sequenced from rabbits collected in the same location that also experienced an RHDV outbreak, while P95 is basal to older Spanish, French and German G1 sequences.

Species determination, achieved by the analysis of nuclear and mitochondrial markers, confirmed the hare specimens as being *L. granatensis* (Table 1), which is also consistent with the morphological characterization and species geographic range in Portugal [4] (Figure 1). Since all five living lagomorph taxa inhabiting the IP (*L. granatensis*, *L. europaeus*, *L. castroviejoi* and both subspecies of *O. cuniculus* – *O. c. cuniculus* and *O. c. algirus*) were represented in these analyses, further confidence is given to the species identification.

*L. europaeus* is the main host of EBHSV, and *L. timidus* is affected only in regions where *L. europaeus* is also present [2]; no reports of EBHSV exist in *L. granatensis* despite the analysis of high numbers of animals (Gortázar C, personal communication). On the IP, *L. europaeus* is restricted to a region between the Ebro River and the Pyrenees, and a narrow contact zone exists between *L. europaeus* and *L. granatensis* [23] (Figure 1). Hence, despite some evidence of ancestral and ongoing hybridization between both species and with *L. timidus* [24], transmission of the virus between these species might be quite limited. In addition, and as for RHDV, the Pyrenees might also restrict EBHSV introduction on the IP. Thus, contact of Iberian hares is more likely to occur with RHDV than with EBHSV.

Our results further show that the RHDV outbreak that occurred at the end of 1997 in a rabbit population, as confirmed by the RHDV positive rabbit samples P141-P144, was the source of the *Lagovirus* that caused the fatal infection in P151. Indeed, P151 was found dead in January-1998, only one month after those rabbits died. The high nucleotide and amino acid identity also confirmed this observation (99.8% and 100%, respectively). The possibility that the observed results were due to contamination was discarded given that RNA extraction, cDNA synthesis and PCR amplification of hare samples were performed independently from the rabbit samples. Also, none of the sequences obtained from hares was identical to previously amplified sequences in our facilities or any other sequences available in public databases. Thus, the susceptibility of Iberian hares (*L. granatensis*) to RHDV is confirmed by our results. Nevertheless, how RHDV is able to overcome species boundaries is unknown.

At present, only glycans of the histo-blood group antigens family have been associated with rabbit susceptibility to RHDV [25]; however, preliminary data indicate that glycans are differently located in hare tissues (Le Pendu J, Abrantes J, Lopes AM, personal communication). Other factors could have promoted the cross infection. The higher mutation capacity of RNA viruses might partially explain their ability to jump species boundaries [26]. Also, the closer the species are to the natural host, the more susceptible they are to the virus [27]. Despite this, the majority of virus transfers to new hosts do not cause severe outbreaks, but only single infections or limited outbreaks [26]. This seems to be the case in our study, where RHDV was able to infect the two hares analysed, but not capable to cause easily detectable outbreaks. Additionally, viral recombination in unfavorable environments is associated with transmission to new hosts [26]. Although recombination is rare in RNA viruses, there are previous reports of recombination in RHDV [28,29]. Screening of our alignment showed no evidence of recombination; nevertheless, only a quarter of the total viral genome was investigated, so it is unclear if recombination contributed to the host switch. Nonetheless, on two occasions, classical RHDV spilled over into individual Iberian hares. While this is an interesting finding, it has not been reported in Iberian hares since then. Therefore, it is likely that these have been isolated “spill-over” events in areas where the habitats of the two hosts overlap and host densities were high [30].

In sum, we report the identification of two different RHDV strains in *L. granatensis* that represent two independent infections, one in 1996 and the other in 1998. This finding of RHDV in Iberian hares represents the earliest evidence of a cross-species transmission of classical RHDV otherwise considered species-specific.
